# Diversity of indigenous sheep of an isolated population

**DOI:** 10.1186/s12917-018-1682-y

**Published:** 2018-11-16

**Authors:** Caroline Marçal Gomes David, Celia Raquel Quirino, Wilder Hernando Ortiz Vega, Aylton Bartholazzi Junior, Aparecida de Fátima Madella-Oliveira, Ricardo Lopes Dias Costa

**Affiliations:** 1 Universidade Estadual do Norte Fluminense, Centro de Ciências e Tecnologias Agropecuária Laboratório de Reprodução e Melhoramento Genético Animal, 2000, Alberto Lamego Ave, Campos dos Goytacazes, Rio de Janeiro, 28016-811 Brazil; 2 Instituto Federal de Educação, Ciência e Tecnologia do Espírito Santo, 482 Rod Br, Km 47, s/n - Rive, Alegre, Espirito Santo 29520-000 Brazil; 3 Instituto de Zootecnia de Nova Odessa, 56, Heitor Penteado St., Nova Odessa, São Paulo, 13460-000 Brazil

**Keywords:** Genetic improvement, Molecular biology, *Ovis aries*, Polymorphism

## Abstract

**Background:**

Because of the influence of genetics on animal production and the risk of losing genetic diversity of naturally adapted breeds, this study evaluated the genetic diversity of sheep of the Morada Nova breed belonging to an animal science institute in Brazil. The herd in question is one of the country’s most representative of the breed. Samples of DNA extracted from the plasma of 61 animals were used for later analysis of the genotypes using microsatellite molecular markers.

**Results:**

The polymorphic information content was 0.66, the observed heterozygosity was 0.65 and the fixation index was 0.048. According to the results, there is moderate genetic diversity in the studied population, suggesting the implantation of breeding programs aimed at conservation of the observed genetic diversity.

**Conclusion:**

The results obtained in this study will be of great importance to decisions on herd structure, besides contributing to other work to be carried out at the research center.

## Background

Morada Nova is one of the main hair sheep breeds in Brazil. They are small animals that stand out among naturalized sheep breeds sheep due to the good production of meat and hides. Because of traits such as high prolificacy [1], parasite resistance [2] and adaptability to edaphoclimatic conditions, this breed is considered useful for crossbreeding, in particular in industrial breeding programs with breeds having higher zootechnical performance. Morada Nova herds are mostly found Brazil’s northeastern semi-arid region, where they are one of the main sources of income of smallholders [3].

Currently, small herds of Morada Nova sheep can also be found in the Southeast and Midwest of Brazil, populations are distributed mainly in research centers, most frequently in São Paulo state. The Instituto de Zootecnia in the municipality of Nova Odessa has one of the main herds representative of the breed, assisting and monitoring producers them in addition to developing research in areas like nutrition, health, reproduction and breeding, among others. Since genetics exerts an influence on production indexes, either positive or negative, so the best use of this potential depends on selection, which in turn requires a diversified number of alleles that can favor larger numbers of combinations.

In addition to support for genetic improvement programs, genetic diversity is a determining factor for the conservation of genetic resources and the evolutionary persistence of species. Naturally adapted breeds may be a source of genetic material able to improve resistance of other breeds to unfavorable conditions [4]. The ability of a population to respond adaptively to environmental changes depends on its level of variability or genetic diversity [5, 6].

Genetic diversity of indigenous breeds is a major concern considering the need to preserve what may be irreplaceable richness regarding new productive demands [7]. A species without enough genetic diversity is thought to be unable to cope with changing environments or evolving competitors and parasites [8]. Conservation should be based on thorough knowledge of the genetic resources of the specific breed [9]. Therefore, it is important to genetically characterize indigenous breeds [9], for which the application of molecular genetics has many important advantages [5].

The aim of obtaining higher animal production rates is impaired by loss of diversity and the introduction of exotic breeds in crosses carried out indiscriminately, modifying the population structure and compromising a breed’s existence. Genetic depression due to inbreeding is also an extremely important factor, also impairing herd performance [10].

To maintain diversity, studies need to be conducted to learn the genetic structure of populations. Microsatellite markers are used to estimate parameters of genetic diversity and herd structure in genetic resource conservation programs due to their dominance and high sensitivity [11].

Considering this, the objective of this work was to evaluate the genetic diversity of sheep of the Morada Nova breed belonging to the Instituto de Zootecnia in Nova Odessa, São Paulo. The results obtained in this study will serve as an aid to future research and conservation of the breed.

## Results

Of the 15 microsatellite markers tested, just INRA63 did not amplify loci and 14 were polymorphic, for a total of 100 detected alleles. We did not observe the presence of null alleles, that is, all samples amplified the 14 microsatellite loci tested. The mean number of alleles (Na) was 7.14 and the mean number of effective alleles (Ne) was 3.68. The locus ILSTS08 presented the lowest polymorphism with two alleles and Ne of 1.44, while locus OARFCB304 was the most polymorphic, with Na 11 and Ne of 7.728.

According to the polymorphic information content (PIC) test, all markers presented PIC above 0.5, considered highly informative, except for the OARAE129 marker, which presented a low PIC of 0.19.

Of the 14 microsatellites tested, seven were in Hardy Weinberg equilibrium with significant *p*-values: *p* < 0.001 (ETH152, INRA06, OARFCB304); *p* < 0.01 (MAF65, OARAE129); and *p* < 0.05 (CSRD24, ILSTS87). Another seven were not in equilibrium, with *p* > 0.05 (ILSTS08, INRA05, INRA172, MCM42, MCM527, OARCP49 and OARFCB20).

In the evaluation of allelic richness (number of different alleles for the same genome region), the mean value of all markers in this population was 4.51, showing the long-term potential of adaptation and persistence of the population. When observed individually by marker, the lowest values of allelic richness were for the loci ILST08 (1.95), OAR129 (2.168) and MCMX42 (3.49), the highest value was in the OAR304 locus (7.06), and the other markers obtained medium values ranging from 4.15 to 5.30.

The mean observed heterozygosity (Ho) in the population was 0.65 while the expected (He) was 0.66. The diversity estimates applied for each locus studied are presented in Table 1.

**Table 1:** Mean number of alleles (Na), effective number of alleles (Ne), observed (Ho) heterozygosity and expected (He), Fixation index (F), allele richness (AR)

Locus	Na	Ne	Ho	He	F	AR
CSRD247	7	3.935	0.721	0.746	0.033	5.04
ETH152	6	4.309	0.656	0.768	0.146	5.19
ILSTS08	2	1.441	0.246	0.306	0.196	1.96
ILSTS87	8	4.731	0.820	0.789	−0.039	5.13
INRA05	10	4.678	0.787	0.786	−0.001	5.30
INRA06	7	4.064	0.705	0.754	0.065	5.04
INRA172	9	2.988	0.705	0.665	−0.060	4.96
MASF65	8	3.832	0.705	0.739	0.046	4.51
MCM42	7	2.612	0.672	0.617	−0.089	3.49
MCM527	7	2.967	0.672	0.663	−0.014	4.15
OARAE129	3	1.265	0.131	0.210	0.375	2.17
OARCP49	8	3.585	0.656	0.721	0.091	4.68
OARFCB20	7	3.510	0.787	0.715	−0.100	4.53
OARFCB304	11	7.728	0.852	0.871	0.021	7.06
Mean	7.14	3.68	0.65	0.66	0.048	4.51
SE	0.63	0.42	0.05	0.05	0.034	1.20

The fixation index (F) reflects the probability that two alleles within the same individual are identical in offspring. Six of the 14 markers tested had negative indices, that is, they were not fixed in the population. For the other markers, two presented moderate indices (ETH152, ILSTS08) and one a high index (OARAE129). The overall mean considering all the microsatellite markers was low (F = 0.048).

The 14 microsatellite loci analyzed showed 91 possible combinations, a total of 16 combinations of markers, the genotypes in one locus are not independent of the genotypes in the other locus. Of the 16 combinations in linkage disequilibrium, only one combination was syntenic (Table 2).

**Table 2:** The linkage disequilibrium between pairs of microsatellites markers

Locus#1	Locus#2	P-Value	SE	LD	Synteny
ETH152	MAF65	0.0000	0,0000	+	N
INRA06	MCM42	0.0000	0,0000	+	N
INRA172	MCM527	0.0028	0,0021	+	N
INRA172	MAF65	0.0029	0,0021	+	N
OARCP49	OARFCB20	0.0037	0,0025	+	N
MCM527	OARFCB20	0.0079	0,0045	+	N
MAF65	MCM527	0.0110	0,0054	+	N
INRA06	MAF65	0.0116	0,0059	+	N
INRA172	OARCP49	0.0131	0,0082	+	N
INRA05	MCM42	0.0162	0,0059	+	N
MCM42	OARFCB30	0.0167	0,0077	+	N
ETH152	OARAE129	0.0189	0,0059	+	S
INRA05	OARFCB30	0.0203	0,0104	+	N
ETH152	ILSTS87	0.0246	0,0097	+	N
CSRD247	OARFCB30	0.0345	0,0157	+	N
MAF65	OARFCB20	0.0371	0,0152	+	N

*P*-Value significance = 0,05. *SE* standard error, *N* Non-syntenic, *S* Syntenic

**Fig. 1:**
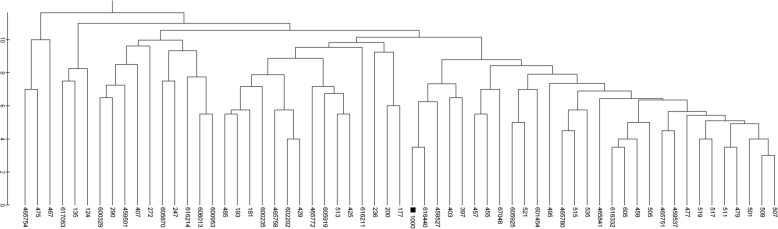
Dendrogram of genetic differentiation between individuals based on Nei’s genetic distance of Morada Nova sheep

The statistical cluster analysis by ascending hierarchical ordering, grouped the 61 animals into two clusters (Fig. 1).

**Fig. 2:**
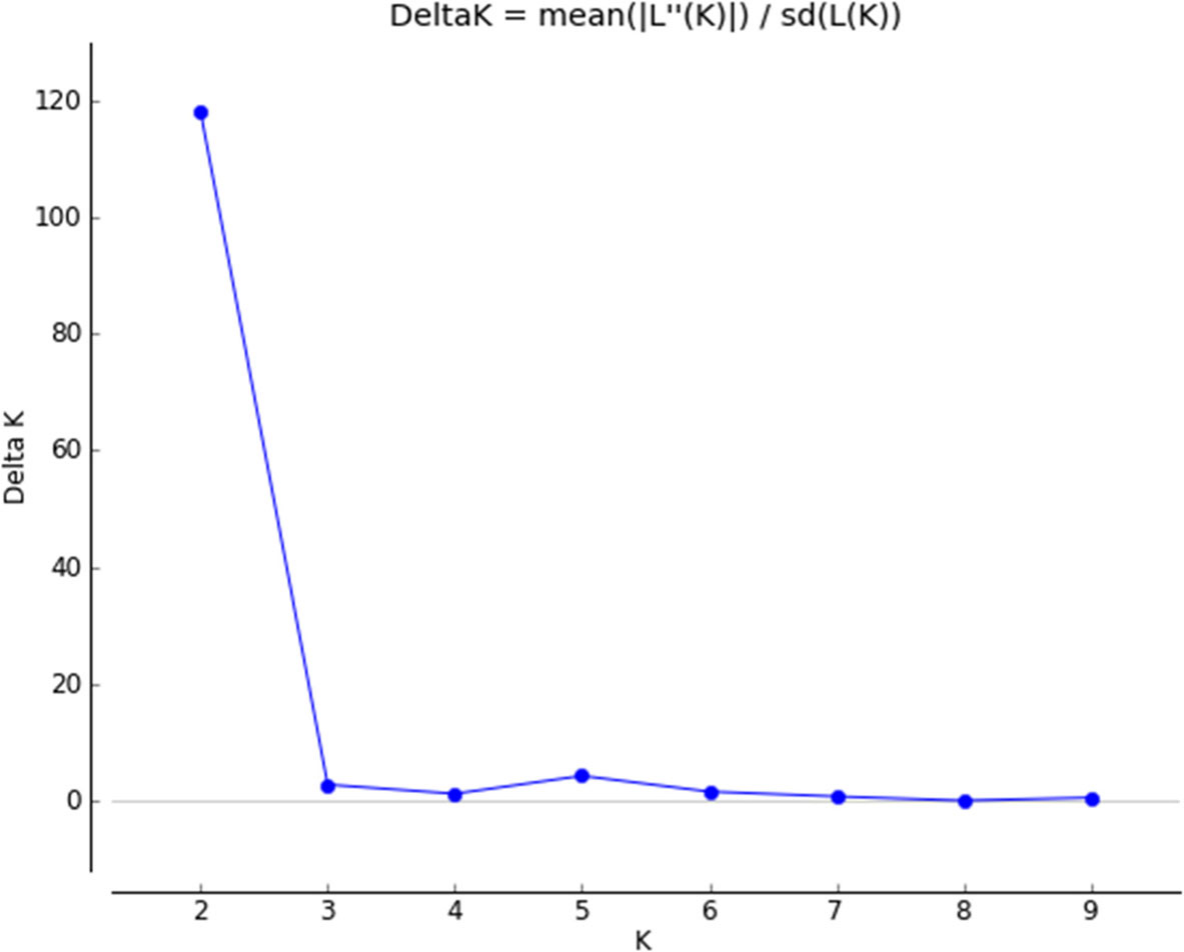
Structure Harvester results (approximate numbers). Graph for the detection of the number of clusters (K) for observation of population group of Morada Nova sheep, realized by the Structure Harvester online software. Delta K values calculated by Evanno’s method detecting K = 2 groups

**Fig. 3:**
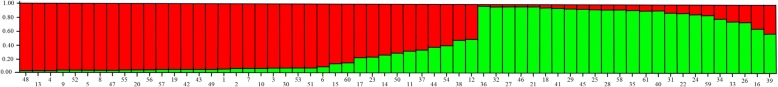
Cluster analysis based on a Bayesian model with 61 representative animals of a single Morada Nova sheep population. The individuals are represented in the vertical bar graph; the colors represent the K genetic cluster; the length of each segment is proportional to the individual’s participation in the cluster; the values on the Y axis show the correlation coefficient. Analysis performed with the STRUCTURE software

Based on the Evanno method for identification of the ideal K in the analysis of population structure (Fig. 2), the individuals were grouped into two populations (K = 2) from the 14 microsatellite markers used in the analysis performed with the Structure software, based on the delineation of clusters of individuals in relation to their genotypes (Fig. 3).

## Discussion

Microsatellite markers are commonly used in paternity tests and are considered an excellent alternative 22 for breed profiling [11, 12], genetic diversity, breeding and conservation programs, [13, 14]. The use of these markers is considered to be reliable because they have characteristics such as high polymorphism, good specificity, high mutation rate and wide genome distribution, besides being easy to analyze [14].

The inter simple sequence repeat (ISSR) technique also presented highly polymorphic loci and has been used previously in genetic diversity studies [9]. These markers are useful to find markers associated with major and minor genes controlling important traits. Several studies have been conducted on associations of ISSR markers with important characteristics, including sheep production traits [7, 15].

Currently, breeding values in genomic selection are generally predicted based on SNP markers (single nucleotide polymorphism). These have also been used in analyses of genetic diversity [16]. There are more than 54,000 validated SNPs for sheep breeds of major economic interest, including Brazilian breeds such as Morada Nova and Santa Inês (Illumina, San Diego, CA). According to Fischer [17], the potential of SNPs to describe the genetic structure of the population depends heavily on the density of the chips used. However, the high cost to purchase these chips restricts research in countries that have low scientific investment, thus justifying preference for the use of microsatellite markers [18]. In studies using a low number of SNPs and microsatellites, the microsatellites presented similar or better results than SNPs [19, 20].

In this study, the microsatellite markers used reflected the genetic diversity evaluated in the population. The 14 markers were highly informative, with PIC above 0.50. PIC values above 0.50 are considered highly, values between 0.50 and 0.25 are moderately informative and values below 0.25 are considered slightly informative [20]. This high observed value is also an indicative parameter for the classification and selection of the microsatellite used, since these presented efficiencies in the detection of polymorphisms. The locus used in this study are commonly used in paternity test and genetic diversity in sheep and are recommended by the International Society for Animal Genetics (ISAG). The linkage disequilibrium observed between the locus is probably due to the inheritance of common alleles of common lineages. According to Flint-Garcia [21], the mating system of the species (selfing versus outcrossing), and phenomena such as population structure can strongly influence patterns of LD.

The number of alleles within a population as one of the parameters of genetic diversity and is a major factor in animal production, since a greater number of alleles within a population allows for greater possibilities of recombination. It is common to note that in commercial or geographically isolated herds, selection pressure exerts forces contrary to diversity, leading to genetic drift. However, the herd studied, although new and composed of small sheep, had a high Na value compared to other studies conducted in different regions analyzing the same breed.

We found 100 alleles, with average number of alleles per locus of (Na) 7.14. The highest number of allele markers were in OARFCB304, with 11 alleles, and INRA05 with 10 alleles, while least number were in ILST08 and OARAE129, with 2 and 3 alleles respectively. This result indicates that even though the population studied is isolated, the Na was higher than that described in other studies evaluating Morada Nova herds. In Ceará, 89 alleles (3.28 alleles per locus) were observed using 17 microsatellite markers in a study performed in 13 different farms [22]. Ferreira [23] used 15 microsatellites, of which, 5 were the same as those studied by us (MAF65, MCM527, INRA172, INRA05, OARF304) in the same breed in a single population, and described Na of 81 alleles and average of alleles 4.62 per locus. Understanding of the relation between the effective size and the real size of a population is of fundamental importance for planning conservation strategies. The mean number of effective alleles (Ne) was 3.68, a medium value considering the value of Na (7.14). The loci with lowest Ne values were also the lowest in the observed heterozygosity. The means were higher than those observed in five distinct populations of Kermani sheep, which presented means of Na 2.94 ± 0.23 and Ne 2.31 + 0.23 [9]. Even though the mentioned study was carried in different populations, the number of animals was low, which showed a loss of diversity of indigenous animals. The authors emphasized that a better understanding of the potential of native species is necessary to support long-term genetic improvement.

Our study revealed the presence of low allelic richness. The decrease of allelic richness may lead to a reduction in the population’s potential for adaptation to future environmental changes, since this diversity is the raw material for evolution through natural selection [17].

The value of 4.51 identified for allelic richness in this study suggests unequal distribution of allelic frequencies with the presence of low-frequency alleles, such as ILST08 (1.96) and OAR129 (2.17). The average of allelic richness observed by GebreMichael [24] was 6.79 for animals of the same breed from different regions. The author observed phenotypic variability among populations and within populations, and attributed this variability as characteristic of traditional populations that were not subjected to a strong selection. However, that author only compared the allelic richness with values reported for other domestic sheep populations, and these values are similar to those observed in our study. On the other hand, the AR values observed by [24] in different breeds were reported to be above 8.0 for six of the seven breeds studied.

Like the AR values, the heterozygosity indices also estimate genetic variation within a population and are one of the most widely used parameters of genetic diversity [25]. Heterozygosity expresses great miscegenation and lower allele fixation. A higher proportion of heterozygous individuals than expected, according to the rate of segregation of a population, is desirable to maintain genetic diversity. The estimates of observed heterozygosity (Ho) and expected heterozygosity (He) in this study (0.65 and 0.67, respectively) represented moderate genetic diversity.

Values of Ho (0.53) and He (0.59) were attributed to the low diversity of the Morada Nova breed [22]. In that study, the authors used animals from eight farms in three different states. That result indicates that the isolated herd used in our study is representative of the breed, with moderate heterozygosity considering the number of animals studied.

Estimates of diversity in populations of sheep from Colombia presented mostly low heterozygosity, with values of Ho below the values of He, with means of 0.68 and 0.77 respectively [26]. In that study, the authors used 513 animals from 13 breeds from 56 farms, and values indicative of diversity were lower than those found in our study. The desired genetic diversity is Ho greater than He. Like the values observed in five Kermani sheep populations in Iran, which presented He of 0.56 ± 0.06 and Ho of 0.97 ± 0.12 [9]. The authors reported enormous biodiversity among domestic animals in developing countries and warned of the loss of this diversity because of the introduction of different breeds and the importance in conserving naturalized breeds.

One of the main factors that causes loss of heterozygosity is the fixation of alleles of commercial interest and mating directed to obtain higher production rates, common in industrial production. This heterozygosity loss increases with rising number of alleles in homozygosis, resulting in allele fixation. According to the FAO (1998), the estimated value through the fixation index (F) should not exceed 1%. In this study, the estimated value of F was 4.8%. The ETH152, ILSTS08 and OARAE markers presented values of 14.6, 19.6 and 37.5, respectively. These large values indicate high homozygous for these loci and possible loss of alleles. Five markers presented values below 6% and another five negative values, with these also being the loci with highest Ho. This fact can be attribuited to the lack of gene flow and rigorous mating control. However, the low number of Morada Nova sheep in this geographic region makes it hard to introduce new genes to increase the diversity.

Likewise, in a study of diversity study of Morada Nova herds located in five Brazilian states including São Paulo, the authors found F values between 4 and 12% and reported consanguinity between the herds [27]. Inbreeding among herds of different regions observed in studies shows that the introduction of animals from different herds alone may not be sufficient to increase the breed’s diversity, so intensified conservation work is important.

The Bayesian group factor analysis performed by the Structure software determined the structure of the sheep population studied and presented a population structure clearly divided into two representative groups, with some individuals presenting a mixture of the two gene groups, possibly due to the miscegenation of the main cluster present. This structure is possibly due to the origin of the herd, which was formed of individuals coming from different states.

The ability of the Bayesian algorithm to detect the most probable number of clusters that explain the distribution of the genetic groups of the population studied was based on a model that correlates the allelic frequencies within the population (admixture model). This contributes to the detection of subpopulations or genetic subgroups in a single population when individuals may be particularly related and presents the same detection ability as the independent allele frequency model, such as the absence of high levels of relationship between the individuals or populations evaluated [28].

The structure of the population presented here is important for the maintenance of genetic diversity through the direction of mating. The dendrogram allows observing the animals according to their genetic distance, which facilitates the identification of the best genetic combinations to avoid endogamous mating.

Diversity is conserved by the maintenance of individuals with few genetic relationships. Knowledge about these relationships helps in the management herds with better use of existing genotypes in breeding programs, besides being fundamental in programs for conservation of genetic resources.

## Conclusion

The herd of Morada Nova of the Instituto de Zootecnia presented moderate genetic diversity. Strategies to maintain or increase genetic diversity should be implemented considering the need to preserve what may be irreplaceable richness and the importance of the herd in the conservation of the breed regarding new production demands, mainly as a maternal lineage in industrial mating.

## Methods

### Locale and population studied

The study was carried out on 61 animals of the Morada Nova breed from the Sheep Unit of the Center for Research and Development of Diversified Animal Husbandry, of the Instituto de Zootecnia, part of the Paulista Agribusiness Technology Agency of the Department of Agriculture, located in Nova Odessa,SP (22 ° 42 ‘S and 47 ° 18’ W).

### DNA extraction and genotyping

DNA was extracted from blood plasma using the commercial NucleoSpin Blood kit (Macherey-Nagel, Düren, Germany) following the manufacturer’s instructions, with subsequent quantification of DNA concentrations measured with a spectrophotometer (NanoDrop™ 2000-Thermo Science). The extraction and purification steps were performed at the Animal Genetic Improvement Laboratory of Norte Fluminense State University in Rio de Janeiro.

Genotyping was performed using a capillary sequencer (MegaBACE 1000 DNA Analysis System - GE Healthcare). We used 15 microsatellite markers recommended for identification of kinship and paternity in sheep by the International Society for Animal Genetics (ISAG): CSRD24, ETH152, ILSTS08, ILSTS87, INRA05, INRA06, INRA172, MASF65, MCM42, MCM527, OARAE129, OARCP49, OARFCB20, OARFCB304 AND INRA 63.

### Statistical analysis

The GenAlEx software version 6.502 was used to evaluate population genetics, to estimate: number of alleles (Na), effective number of alleles (Ne), observed heterozygosity (Ho), expected heterozygosity (He), fixation index (F); and polymorphic information content analysis (PIC). Linkage disequilibrium was estimated for all marker pairs using GenePop software version 1.2 [29].

The distribution of genetic diversity was studied using the genetic distance analysis of Nei. For the Hardy-Weinberg equilibrium (HWE), Fisher’s exact test was applied. The population allele richness (A_R_) measure of the 14 markers was estimated using the HP-RARE 1.0 software. Using Nei’s genetic distance matrix with standardized data the dendrogram was constructed from the unweighted pair group method with arithmetic mean using the software MEGA-5 (Molecular Evolutionary Genetics Analysis).

The population structure was analyzed using the software STRUCTURE 2.3.4, which employs functions based on Bayesian clustering algorithms, considering a mixed ancestry model (admixture model) and correlating the allelic frequency in the population. The burn-in period was 200,000 rounds followed by 500,000 Monte Carlo Markov Chain interactions. To observe numbers of possible clusters in the population, the independent “K” test was performed from 1 to 10 clusters with 20 replicates, thus verifying the consistency of the results. The ideal “K” value was evaluated after the analysis of the result file by the Evanno method using the web-software STRUCTURE Harvester version 0.6.94.2012 software. After identification of the number of subpopulations, a test was performed to select the optimal “K” to generate the illustrative population plot.

## Abbreviations

Ar: Allele richness
F: Fixation index
He: expected heterozygosity
Ho: observed heterozygosity
HWE: Hardy-Weinberg equilibrium
ISAG: International Society for Animal Genetics
ISSR: Inter simple sequence repeat
LD: Linkage disequilibrium
MEGA-5: Molecular Evolutionary Genetics Analysis
Na: Number of alleles
Ne: effective number of alleles
PIC: Polymorphic information content
